# ImputeGAN: Generative Adversarial Network for Multivariate Time Series Imputation

**DOI:** 10.3390/e25010137

**Published:** 2023-01-10

**Authors:** Rui Qin, Yong Wang

**Affiliations:** School of Computer Science and Engineering, University of Electronic Science and Technology of China, Chengdu 611731, China

**Keywords:** data imputation, GAN, informer

## Abstract

Since missing values in multivariate time series data are inevitable, many researchers have come up with methods to deal with the missing data. These include case deletion methods, statistics-based imputation methods, and machine learning-based imputation methods. However, these methods cannot handle temporal information, or the complementation results are unstable. We propose a model based on generative adversarial networks (GANs) and an iterative strategy based on the gradient of the complementary results to solve these problems. This ensures the generalizability of the model and the reasonableness of the complementation results. We conducted experiments on three large-scale datasets and compare them with traditional complementation methods. The experimental results show that imputeGAN outperforms traditional complementation methods in terms of accuracy of complementation.

## 1. Introduction

Time series data are widely available and useful in many fields, such as medicine [1], economics [2], and traffic forecasting [3]. The quality of the data largely determines the quality of downstream tasks. However, device failure and network connectivity problems inevitably occur during the use of IoT devices. This can bring about the problem of missing data. Obviously, the lack of data can have a significant impact on the subsequent analysis and utilization of the data [4]. The authors in [5] attempted to predict the groundwater level in a well field using artificial neural networks (ANNs) and support vector machines (SVMs). When used for the prediction of missing data, an ANN is not as effective as an SVM. However, when the missing data are found, an ANN predicts better than an SVM. When complete data are used, both ANN and SVM predict better than when incomplete data are used.

Complementary methods for missing data can be roughly divided into two categories, statistical and neural-network-based complementary methods. Statistical methods include mean value imputation [6], last value imputation [7], mode value imputation [8], K-nearest neighbor (KNN) algorithm-based imputation [9], and matrix factorization algorithm-based imputation [10]. In addition to this, there are many other approaches, including SSMLP [11], extensions to KNN [12], RENUVER [13], and Holoclean [14]. These methods are effective, but difficult to apply to multivariate time series data because good feature engineering is required for them to work, and feature engineering, in turn, requires experts to perform analysis. Therefore, these methods cannot be used for end-to-end models.

Neural-network-based complementation methods include recurrent neural network (RNN)-based complementation methods [15,16,17,18,19] and generative adversarial network (GAN)-based complementation methods [18,19,20]. Compared with statistical complementation methods, GAN-based complementation methods can effectively utilize the temporal information of data [18]. Compared with RNN-based complementation methods, GAN-based complementation can more adequately determine whether complementation results are close to the true results because GAN generates missing data via a generative method that are judged by a discriminator, while RNN completes the missing values by fitting. Whenever a complementary dataset of a GAN used for complementation is changed, it needs to be retrained. We ensured the generalizability of the GAN-based complementation method by designing a new complementation network architecture. An RNN network suffers from the problem that positions far from the current input do not have enough weight, which causes the initial input to fail to impact the output when the sequence is too long [21]. A transformer [22] solves this problem with positional embedding. The proposed informer [23] not only reduces the memory consumption and time consumption of transformers in long-term time series forecasting (LSTF), but it also improves the accuracy of forecasting. Moreover, the current GAN-based complementation method does not further iterate and optimize the complementation results. To overcome this problem, we propose a new iterative strategy to compare the strengths and weaknesses of the complementation results. The method first trains an autoencoder that is capable of converting the data into appropriate feature vectors on a small number of missing datasets. Afterwards, the missing data are fed into this autoencoder to obtain the feature vectors corresponding to the missing data. Then random vectors are generated, and a gradient descent method is used to make these random vectors correspond to the generated data as close as possible to the real data. Lastly, the vector that is closest to the real data is selected from these vectors as the complementary result. Experiments on real datasets demonstrate that our approach achieves a better imputation accuracy and better time consumption compared to those of other methods.

Our contributions can be summarized as follows:

- We propose a GAN-based neural network for complementation that uses a generative approach to generate complementary data and judge them with a discriminator that can handle continuously missing data more effectively.
- In contrast to other GAN-based methods of complementation, imputeGAN ensures that the complementation values are reasonable through an iterative strategy.
- The generalizability of the model is ensured with a carefully designed training framework.

## 2. Related Work

Missing data can be classified into three types: missing completely at random (MCAR), missing at random (MAR), and missing not at random (MNAR) [24]. MCAR implies that the data are missing completely at random, do not depend on a missing or observed variable, and do not affect the unbiasedness of the sample. MAR means that the probability of missing data is not related to the missing data themselves, but only to the partially observed data. MNAR implies that missing data are related to the values of the incomplete variables themselves and the observed variables.

MCAR, the case deletion method of removing the cases with missing terms, is an option. This is because removing missing cases does not affect the analysis of the overall data under this condition. However, when there are too many missing cases, the case deletion method leads to a significant reduction in the amount of information available for analysis. In addition, when dealing with MAR, the method loses the information related to the missing data in the residual data [24]. Obviously, imputing the missing values with the most probable values produces less information loss than deleting incomplete samples does. Traditional data completion methods fill in the missing data with a mean value [6], last value [7], or mode values [8]. While these methods can quickly fill in missing values, they can affect the variance in the original data. For example, if a missing value is numeric, the missing attribute is filled with the average of the values of the attribute in all other objects; if the missing value is non-numeric, the missing attribute is filled with the value of the attribute with the highest number of values in all other objects. So, this replaces the features of the overall data with the features of part of the data, which can introduce bias even if the missing type of the data is MCAR [24]. Common machine-learning-based complementary methods include k-nearest neighbor methods, recurrent neural networks, generative adversarial networks, and matrix factorization algorithms. The K-nearest neighbor (KNN) [9] algorithm fills missing values with the weighted average values in the nearest k neighbors of the missing data. K neighbors are selected on the basis of some distance metric, and their weighted mean values are used to interpolate the missing data. This method requires us to choose the value of k and the distance metric. Compared with the case deletion method, KNN does not have to remove missing cases, which allows for it to preserve the characteristics of the time series data [25]. The KNN imputation method, implemented by Chen and Chiu [26], renders the mean absolute error (MAE) and root mean squared error (RMSE) between the complementation result and the true result as small as possible. One obvious drawback of the KNN algorithm is that it becomes very time-consuming when analyzing large datasets because it searches for similar data points throughout the dataset. In addition, in high-dimensional datasets, the difference between the nearest and farthest neighbors is very small, so the accuracy of KNN is reduced. The matrix factorization [10] algorithm imputes data by decomposing a time series matrix into the product of two lower-dimensionality rectangular matrices, and uses the product of these matrices for missing values. When dealing with MNAR, these methods often require additional processing to be able to fill in the data more accurately [27].

Random forest is an efficient way to fill in missing data [28]. However, when the missing data are multivariate time series data, feature engineering is required before this method can be used. The feature engineering of time series data includes the analysis of whether the timestamp is a special time, taking the past timestamp for the feature analysis of the current timestamp. When analyzing multivariate time series data, the above feature engineering needs to be performed for each variable. This is very time-consuming; thus, it is difficult to form an end-to-end model. In contrast, neural-network-based models do not require additional processing for multivariate data.

The RNN-based complementary method [15,16,17] completes the missing data by fitting the input. RNN-based data-completion models can capture the temporal dependencies of time series data [29], but additional processing is required if the relationships between missing variables need to be learned [16]. Since BRITS speaks of missing data as variables, M-RNN treats missing data as constants. This indicates whether the corresponding data update the gradient during the gradient update. That is, M-RNN ignores the effect of the relationship between missing variables.

The problem with RNNs is that the weights of the current inputs become negligible after some time, but they should not be ignored [21]. Since GAN is a generative model, it does not have this problem. GAIN [20] is a GAN-based imputation model. The generator in GAIN observes real data vectors and imputes missing data, and the discriminator determines which part of the vectors were imputed. As mentioned above, the goal of using GAN is to make the generator generate data with the same distribution as the original data through the game between the generator and the discriminator. The GAIN generator updates the gradient by comparing the difference between the complemented data matrix and the original data matrix. The discriminator of GAIN calculates the probability that each position of the complementary matrix is a complementary value by using the complementary data matrix and the hint matrix. The hint matrix guarantees a random value of 0 or 0.5 for the missing positions and a value of 1 for the non-missing positions. GAIN has greatly improved the accuracy of data imputation. Both GAN-2-stage and E2GAN use the architecture of GAN with RNN as the feature extractor in the generator and discriminator. They train the generator to learn the original input distribution by adding noise to the input and denoising it.

However, these methods are not further optimized for complementary results. For these complementation methods, the correctness of these different sequences is the same. However, in fact, the similarity of these complementation results to the real results is different. Therefore, we propose a new iterative method to iterate over the complementary results. The iterated results are made to be as close as possible to the true results by a carefully designed loss function.

The above comparison is summarized in Table 1. As shown in Table 1, “generative” means that the method uses a generative approach rather than a fitted approach to complete the data, which is more effective for long-term continuous missing data. “Iterative” means that the method iterates and selects the few results that are completed to ensure that the iterated results are closer to the true values.

Time series data forecasting can be divided into traditional and machine-learning-based methods. The former include the ARIMA model [30], the Holt model [31] for data without obvious trends or seasonal factors, and the Prophet model [32], which does not require the insertion of missing values for forecasting. The latter are mainly based on recurrent neural networks and their variants for time series data forecasting [21,33,34]. However, when these methods are applied to the complementation of missing data, they do not work well. Our downstream work is to improve the accuracy of completing missing time series data.

## 3. Preliminary

**Generative Adversarial Networks (GANs)**. GANs [35] consist of generators and discriminators, and train generators that can generate data with the same distribution as that of the original data by gaming the discriminators with the generators. The role of the generator is to map the n-dimensional vector into the data in the sample space. The role of the discriminator is to determine whether the samples are from the original dataset or from the data generated by the generator. The loss function for GANs is as follows.

(1) $$\min_{G}\max_{D}V\left(D,G\right)=E_{x∼p_{data}\left(x\right)}\left[\log D\left(X\right)\right]+E_{z∼p_{z}\left(z\right)}\left[\log\left(1-D\left(G\left(z\right)\right)\right)\right]$$

Equation (1) is divided into $\min_{G}$ and $\max_{D}$. When training the discriminator, the corresponding loss function is shown in Equation (2).

(2) $$\max_{D}V\left(D,G\right)=E_{x∼p_{data}\left(x\right)}\left[\log D\left(X\right)\right]+E_{z∼p_{z}\left(z\right)}\left[\log\left(1-D\left(G\left(z\right)\right)\right)\right]$$

The discriminator is expected to accurately determine the original input and the generator input, i.e., D(X) approximates to 1 and D(G(z)) approximates to 0, thus making Equation (2) converge to 0. Similarly, when training the generator, the corresponding loss function is shown in Equation (3).

(3) $$\min_{G}V\left(D,G\right)=E_{x∼p_{data}\left(x\right)}\left[\log D\left(X\right)\right]+E_{z∼p_{z}\left(z\right)}\left[\log\left(1-D\left(G\left(z\right)\right)\right)\right]$$

It is expected that the trained discriminator is unable to determine the output of the generator, i.e., D(G(z)) approximates to 1, thus making Equation (3) converge to the minimal value. This game process is repeated until the ideal generator is trained.

**Data Imputation**. For *d*-dimensional time series data *x* being recorded at time $t=(t_{1},t_{2},\cdots ,t_{n})$, they can be expressed as $x=\left(x_{1},x_{2},\cdots ,x_{n}\right)\in R^{d\times n}$. $x_{i}$ is the recorded value at time $t_{i}$ since the information of missing data locations is important for data imputation. We recorded this information with the corresponding mask $m=\left\{m_{1},m_{2},\cdots ,m_{n}\right\}\in R^{d\times n}$. If there is missing part in $x_{t}$, the corresponding part is set to 0 and vice versa. Multivariate time series data $X_{i}$ and the mask matrix $M_{i}$ are shown in Equation (4).

(4) $$X_{i}=\left[\begin{array}{cccc} 1 & / & 3\\ / & 5 & / & \cdots \\ 7 & 8 & / \end{array}\right]M_{i}=\left[\begin{array}{cccc} 1 & 0 & 1\\ 0 & 1 & 0 & \cdots \\ 1 & 1 & 0 \end{array}\right]$$

Our goal is to achieve the accurate prediction of multivariate long time series with incomplete data input.

## 4. Proposed Method

Our method completes the missing data by training an autoencoder on datasets with a small number of missing data, and completes the missing data on the basis of this autoencoder. The encoder is used to extract the corresponding feature vectors from the original data, and the decoder is used to recover the corresponding data on the basis of feature vectors. In the actual use of the model, we input the missing data into the encoder and randomly generate several vectors that are passed through the decoder to generate the time series data. We used the gradient descent method to iterate over these vectors to minimize the difference between the time series data generated by the decoder and the missing data. The vector with the smallest difference between the generated data and the missing data is selected, and the result of this vector generated by decoder is used as the complementary result of the missing data. Since the decoder is trained on a small number of missing datasets, we expect it to learn the variation patterns implied in the original data. We formally introduce the method in a later section.

**Figure 1:**
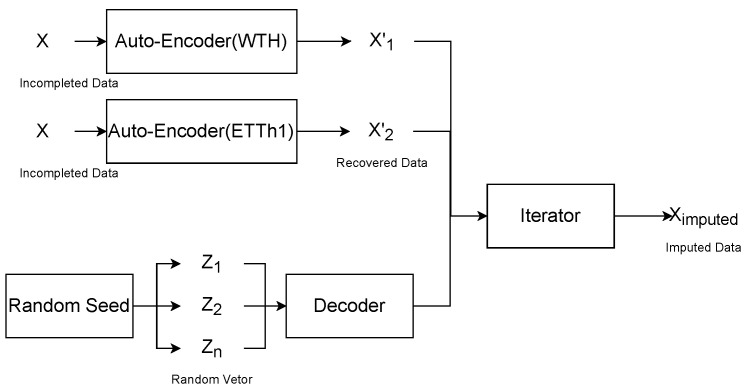
The overall architecture of the proposed imputation approach. The self-encoder can transform incomplete time series data into recovered data, and the randomly generated vectors are decoded into time series data by the decoder, and the difference values between these data are compared. The gradient descent method is used to iteratively update the random vector, and the random vector with the smallest difference between the generated and recovered data is taken as the feature vector of the complementary value, and the corresponding data are the complementary data.

As shown in Figure 1, the corresponding self-encoders are first trained on different datasets. The encoder of the self-encoder extracts the feature vector of the input data and the decoder reduces the feature vector to the full vector. The randomly generated vectors are then decoded by the decoder as the input to participate in the iterations. The gradient of the input is updated using the gradient descent method, and the vector with the smallest difference from the recovered data is taken as the result of the recovery.

### 4.1. Encoder Network Architecture

As mentioned before, the encoder needs to extract the corresponding feature vectors from the original time series data. We adopted the informer’s encoder as the encoder of the autoencoder. We trained multiple encoders on different datasets to ensure generalizability, and used multiple encoders to extract features from the same time series data when completing the data. By giving the feature vector enough initialization space, this ensures that the final iterated feature vector is as close to the true value as possible.

**Figure 2:**
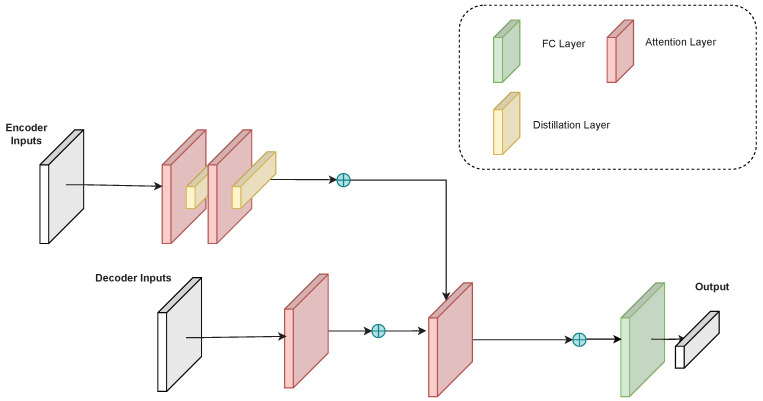
Overall informer architecture. The encoder takes the incomplete time series input and compresses it into the corresponding feature vectors. The decoder takes the compressed feature vectors and outputs the time series data generated from the feature vectors.

As shown in Figure 2, the encoder extracts the feature vectors from the input through the attention layer, removes the redundant information through the distillation layer and repeats the process several times. The encoder of the informer can be formulated as follows:

(5) $$Z_{i\_enc}^{0}=Distilling\left(Attn\left(X_{i\_enc}\right)\right)$$

(6) $$Z_{i\_enc}^{j+1}=Distilling\left(Attn\left(Z_{i\_enc}^{j}\right)\right)$$

(7) $$features_{i\_enc}=Z_{i\_enc}^{last}$$

where $X_{i\_enc}$ denotes the incomplete time series data.

### 4.2. Decoder Network Architecture

The role of the decoder is to recover the vector into time series data. Similar to the encoder, we also used the informer’s decoder as the decoder of the autoencoder. Since we had trained multiple encoders before, we have to train the corresponding multiple decoders. When complementing the data, the data recovered by the decoder are compared with the original data, and the data with the smallest gap are selected with the corresponding vector.

As shown in Figure 2, the decoder extracts the feature vector from the input through the attention layer and recovers the complementary data from the vector through the fully connected layer. The decoder of the informer can be formulated as follows:

(8) $$Z_{i\_dec}^{0}=Attn\left(X_{i\_dec}\right)$$

(9) $$Z_{i\_dec}^{j+1}=Attn\left(Z_{i\_dec}^{j}\right)$$

(10) $$outputs=FC(Attn\left(features_{i\_enc},Z_{i\_dec}^{last}\right))$$

where $X_{i\_dec}$ denotes the second half of $X_{i\_enc}$ with an all-zero mask of imputation length.

### 4.3. Iteration Strategy

When the training of the encoder and decoder of the autoencoder is completed, we input the incomplete data *X* into encoder *E* to obtain the corresponding feature vector *x* and randomly generate the feature vector *z* with the same dimension as *x*. Multiple iterations were performed to find the vector z∗ that minimizes the following equation.

(11) $$\min_{z}{∥G\left(z\right)-X^{\prime }⊕M∥}_{2}^{2}$$

where *M* is the mask matrix identifying the missing data. Then, z∗ was input into decoder *D* to obtain the corresponding time series data Z∗. By randomly selecting *n* initial vectors and performing *L* iterations of gradient descent, we were able to estimate z∗, satisfying the condition as much as possible.

**Figure 3:**
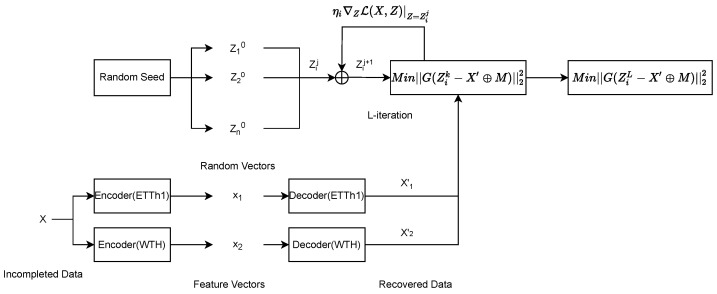
Iterating through the n imputation results to find the optimal imputation result. The same missing data are recovered differently by different autoencoders, but their nonmissing parts are the same. By randomly generating vectors and iterating them, the decoded data of the random vector are rendered as consistent as possible with the nonmissing part of the recovered data. The result with the smallest difference between the decoded and recovered data in the nonmissing part is found as the result of the complementation.

As shown in Figure 3, n is the number of randomly selected vectors $Z_{i}^{0}$, the stochastic gradient descent method is used to iterate $Z_{i}$ for L times, so that the time series data generated by $Z_{i}$ have the smallest possible difference from $X^{\prime }⊕M$. Then the vector $Z_{i}^{L}$ with the smallest gap with $X^{\prime }⊕M$ among these n vectors is taken as the feature vector of the optimal imputation value. The formulation of iteration strategy as follows.

(12) $$Z_{i}^{j+1}=Z_{i}^{j}+\eta_{i}\nabla_{Z}{L\left(X,Z\right)|}_{Z=Z_{i}^{j}}$$

(13) $$L\left(X,Z\right)={∥G\left(Z\right)-X^{\prime }⊕M∥}_{2}^{2}$$

where $\eta_{i}$ denotes the learning rate.

### 4.4. Imputation Results

For each incomplete time series datum *X*, its complementary result is derived from the corresponding part of its reconstruction vector $X^{\prime }$. Thus it can be expressed by the following equation.

(14) $$X_{imputed}=X⊙M+X^{\prime }⊙\left(1-M\right)$$

### 4.5. Discussion

When GAN is used to fill in missing values in time series data, it simply maps random vectors to complete time series data. Therefore, the difference of random vectors can have a great impact on the generated results. Existing approaches ensure that the generated results are close to the true results by adding a penalty term to the loss function of the generator that measures the difference on the nonmissing values. In contrast, imputeGAN makes the complementary results more reasonable by iterating over the generator’s complementary results. The iterative process is described in Section 4.3.

Comparing the differences in the time efficiency of GANs is difficult because they have different parameter settings and training methods. In the case of E2GAN, for example, to ensure the effectiveness of the generator, the generator has to be trained several times for each training of the discriminator. In addition, the size of the dataset used for training has a significant impact on the training time. ImputeGAN uses multiple datasets in the training process in order to ensure the generalization of the model, which leads to its time disadvantage compared to other GAN-based methods.

## 5. Experimental Evaluation

In this section, we present the results of our proposed model run on real datasets and compare them with the baseline.

### 5.1. Datasets, Tasks, and Baseline

We evaluated our proposed model on four real datasets: two medical datasets, an electricity transformer temperature dataset, and a city weather dataset.

**Electricity Transformer Temperature** (ETT dataset was acquired at https://github.com/zhouhaoyi/ETDataset (accessed on 22 June 2022).) (ETT): The ETT dataset contains two years of data on oil temperature of power transformers in two counties in China and six other metrics.

**KDD Cup 2018 Dataset** (KDD CUP. Available on: http://www.kdd.org/kdd2018/ (accessed on 22 June 2022), 2018.) (KDD): contains weather data and air pollution data collected hourly from 30 January 2017 to 30 January 2018 for Beijing and London.

**Electricity Consuming Load** (ECL Dataset. Available on: https://archive.ics.uci.edu/ml/datasets/ElectricityLoadDiagrams20112014 (accessed on 22 June 2022).) (ECL): contains electricity consumption data for 321 clients from 2012 to 2014. The dataset has a time interval of hours and 1% missing data.

**Weather Dataset** (Weather Dataset. Available on: https://www.ncei.noaa.gov/data/local-climatological-data/ (accessed on 22 June 2022).) (Weather): contains weather data collected hourly from 2010 to 2013 for 1600 locations in the United States. The dataset has 5% missing data. A comparison of the datasets is shown in Table 2. The second column of Table 2 indicates the number of features of the time series data, the third column indicates how many moments of data were collected in total, the fourth column indicates the missing rate of the dataset, and the fifth column indicates the interval time of the time series data.

**Downstream Task**: After we had completed training on a dataset with a small number of missing data, we took the data in the test set, discarded 50% of it at random, and attempted to complete the dataset. The accuracy of our method was compared with the accuracy of the prediction method on the basis of the original dataset to measure the effectiveness of our method when used for prediction on the missing dataset.

The baseline models are as follows.

- **Statistical imputation methods**: we filled in the missing values with the average [6] or the last observed value [7].
- **LSTnet** [36]: uses a CNN and an RNN to predict time series data.
- **LSTMa** [37]: adds an automatic search strategy to the encoder–decoder architecture.
- **Reformer** [38]: improves transformer efficiency by locally sensitive hashing self-attention.
- **LogTrans** [39]: the LogSparse transformer improves transformer efficiency by using a heuristic method.
- **Informer** [23]: improves transformer efficiency with ProbSparse self-attention.

### 5.2. Implementation Details

In our experiments, the encoder stack of the informer in the generator contained a stack of three attention layers with two distillation layers. the decoder stack contained two attention layers. The dropout rate was fixed at 0.05. All datasets were normalized, and missing values were padded with 0. Then, 10% of the dataset was used as the validation set, and another 10% was taken as the test set. The optimizer was the ADAM optimizer.

### 5.3. Performance Comparison of Downstream Task

Table 3 shows the results of our proposed method compared with other prediction methods. Our method was used to complete the dataset with 50% of the data missing, the prediction method was used to predict the data on the basis of the original data, and the mean fill was used to fill the missing values on the basis of the mean value of the data at a certain time before. The first column of the table represents the dataset, the second column represents the step size used to fill or predict, the first row represents the method to fill or predict, and the second row represents the measure of the difference between the predicted or filled value and the actual value. The average column indicates that the fill method was based on the average of the data before the missing values. The $MSE=\frac{1}{n}\sum_{i=1}^{n}{\left(y-\hat{y}\right)}^{2}$ and $MAE=\frac{1}{n}\sum_{i=1}^{n}\left|y-\hat{y}\right|$ are used to indicate the difference between the imputation values and the actual values. The smaller these values are, the better the imputation is. In the case of 50% missing data, the accuracy of our imputation method still does not lag too far behind.

### 5.4. Analysis

**Influence of Missing Rate**

**Figure 4:**
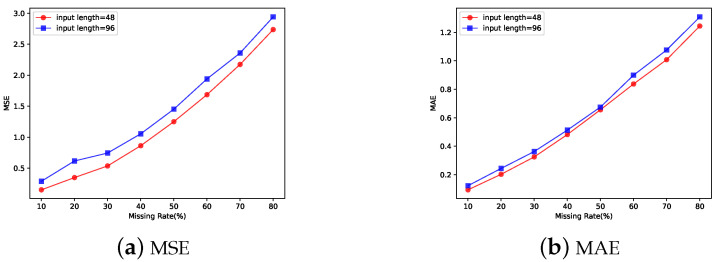
Influence of missing rate on the accuracy of the imputation. (**a**,**b**) The accuracy of complementation gradually decreased with the increase in missing data. In addition to this, increasing the length of the sequences used for completeness decreases the accuracy of completeness.

We also investigated the effect of the missing rate of the input time series data on the accuracy of the imputation.The results are shown in Figure 4. Figure 4a,b depict the effect of the missing rate of the data on the MSE versus MAE of the fill results when other conditions are held constant. They illustrate that the length of the input also affects the MSE and MAE of the imputation when the missing level is the same. As the missing rate of the dataset increases, the accuracy of the imputation decreases accordingly.

**Influence of Datasets**

**Figure 5:**
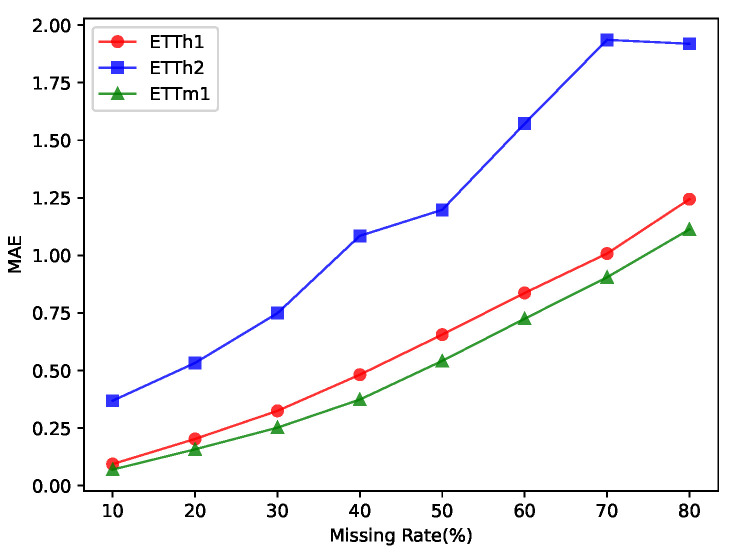
Influence of datasets on the accuracy of the imputation. The increase in the missing rate of the original dataset led to a decrease in the accuracy of data completion.

We also investigated the effect of the input time series data on the accuracy of the imputation. The results are shown in Figure 5, where the x axis indicates the missing rate of time series data, and labels indicate the name of the time series data. The accuracy of our method for completing was high when the original dataset was missing to a small extent, but when there was no sufficiently complete missing dataset, the accuracy of our method for completing suffered when it was used.

**Ablation Study**

**Figure 6:**
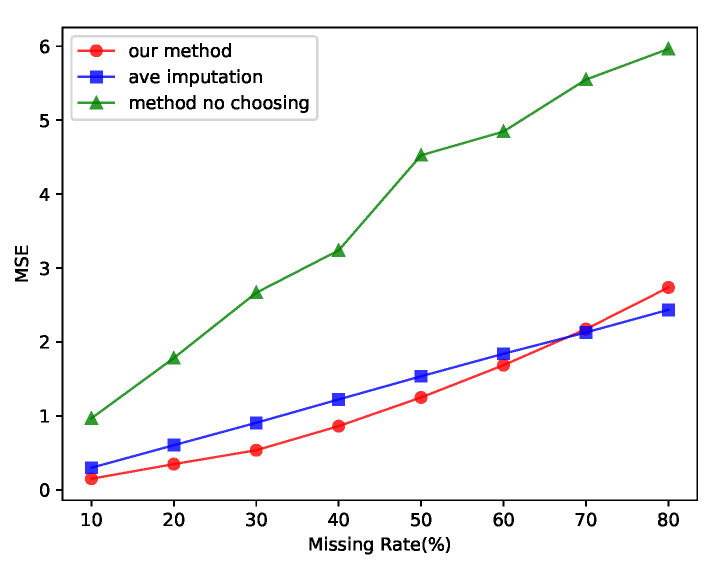
Ablation study of our method without a discriminator and choosing strategy. Our method had better complementary results than those of the mean fill method in most cases.

We tested our proposed method on the ETTh1 dataset and the method of filling the missing values with the mean value and the method of de-selection strategy for the results. The results of the tests are shown in Figure 6, in which the x axis shows the missing rate of the dataset, the line marked with our method shows the test results of our proposed method, the line marked with ave imputation shows the method of filling the missing values with the mean value, and the line marked with method-no-choosing shows our method with the selection strategy for the results removed. Only when the degree of missing data was extremely high did the method of filling with the average value work better than our proposed method.

## 6. Conclusions

In this paper, we proposed a GAN and informer-based model called imputeGAN for solving missing multidimensional time series data.The model was compared with traditional complementary methods and models used to predict time series data. Experimental results demonstrate that imputeGAN outperformed traditional methods in terms of accuracy. When performing downstream tasks such as time series data prediction, even with 50% of the original data missing, the accuracy of imputeGAN complementation was similar to that of models that perform prediction with complete data (e.g., LSTnet). We proposed a multidimensional time series data imputation model based on GAN and Informer. We proposed a new method for the selection and iteration of data complementation results, which makes the complementation results closer to the true values. By comparing imputation and downstream tasks on four real datasets, we confirmed that our new model had better imputation results than those of existing models.

## Figures and Tables

**Table 1:** Comparison of imputation methods.

	MCAR	MAR	MNAR	Generative	Iterative
Mean/last/mode imputation	✓	✓	✕	✕	✕
KNN	✓	✓	✕	✕	✕
Matrix factorization	✓	✓	✕	✕	✕
BRITS	✓	✓	✓	✕	✕
M-RNN	✓	✓	✓	✕	✕
GAIN	✓	✓	✓	✓	✕
GAN-2-stage	✓	✓	✓	✓	✕
$E^{2}$GAN	✓	✓	✓	✓	✕
imputeGAN	✓	✓	✓	✓	✓

**Table 2:** Dataset details.

Dataset	Features	Samples	Missing Rate	Interval Time
ETTh1	7	17,420	1%	1 h
ETTh2	7	17,420	10%	1 h
ETTm1	7	69,680	1%	15 min
ECL	321	26,280	1%	1 h
Weather	12	35,040	5%	1 h

**Table 3:** MSE and MAE results of imputation and prediction methods on five datasets.

Methods		Informer		LogTrans		Reformer		LSTMa		LSTnet		Our Method		Average	
Metric		MSE	MAE	MSE	MAE	MSE	MAE	MSE	MAE	MSE	MAE	MSE	MAE	MSE	MAE
ETTh1	24	0.577	0.549	0.686	0.604	0.991	0.754	0.650	0.624	1.293	0.901	1.250	0.656	1.536	0.744
	48	0.685	0.625	0.766	0.757	1.313	0.906	0.702	0.675	1.456	0.960	1.452	0.675		
	168	0.931	0.752	1.002	0.846	1.824	1.138	1.212	0.867	1.997	1.214	1.491	0.636		
	336	1.128	0.873	1.362	0.952	2.117	1.280	1.424	0.994	2.655	1.369	1.996	0.659		
	720	1.215	0.896	1.397	1.291	2.415	1.520	1.960	1.322	2.143	1.380	2.818	0.769		
ETTh2	24	0.720	0.665	0.828	0.750	1.531	1.613	1.143	0.813	2.742	1.457	5.288	1.198	3.071	1.005
	48	1.457	1.001	1.806	1.034	1.871	1.735	1.671	1.221	3.567	1.687	5.835	1.312		
	168	3.489	1.515	4.070	1.681	4.660	1.846	4.117	1.674	3.242	2.513	5.732	1.236		
	336	2.723	1.340	3.875	1.763	4.028	1.688	3.434	1.549	2.544	2.591	7.375	1.327		
	720	3.467	1.473	3.913	1.552	5.381	2.015	3.963	1.788	4.625	3.709	9.934	7.705		
ETTm1	24	0.323	0.369	0.419	0.412	0.724	0.607	0.621	0.629	1.968	1.170	0.909	0.542	1.527	0.740
	48	0.494	0.503	0.507	0.583	1.098	0.777	1.392	0.939	1.999	1.215	0.977	0.55		
	96	0.678	0.614	0.768	0.792	1.433	0.945	1.339	0.913	2.762	1.542	1.068	0.575		
	288	1.056	0.786	1.462	1.320	1.820	1.094	1.740	1.124	1.257	2.076	1.458	0.690		
	672	1.192	0.926	1.669	1.461	2.187	1.232	2.736	1.555	1.917	2.941	1.375	0.659		
Weather	24	0.335	0.381	0.435	0.477	0.655	0.583	0.546	0.570	0.615	0.545	3.750	0.858	4.531	1.213
	48	0.395	0.459	0.426	0.495	0.729	0.666	0.829	0.677	0.660	0.589	3.438	0.854		
	168	0.608	0.567	0.727	0.671	1.318	0.855	1.038	0.835	0.748	0.647	4.120	0.884		
	336	0.702	0.620	0.754	0.670	1.930	1.167	1.657	1.059	0.782	0.683	3.906	0.861		
	720	0.831	0.731	0.885	0.773	2.726	1.575	1.536	1.109	0.851	0.757	3.997	0.912		
ECL	48	0.344	0.393	0.355	0.418	1.404	0.999	0.486	0.572	0.369	0.445	1.118	0.619	5.993	1.498
	168	0.368	0.424	0.368	0.432	1.515	1.069	0.574	0.602	0.394	0.476	1.22	0.643		
	336	0.381	0.431	0.373	0.439	1.601	1.104	0.886	0.795	0.419	0.477	1.205	0.622		
	720	0.406	0.443	0.409	0.454	2.009	1.170	1.676	1.095	0.556	0.565	1.352	0.664		
	960	0.460	0.548	0.477	0.589	2.141	1.387	1.591	1.128	0.605	0.599	1.002	0.571		

## Data Availability

The code used in this article is available at https://github.com/ubikpkd/d-gan_informer. A reproduction of the code provides access to all of the data generated in this paper.
